# Trends of antibiotic use at the end-of-life of cancer and non-cancer decedents: a nationwide population-based longitudinal study (2006–2018)

**DOI:** 10.1017/ash.2024.75

**Published:** 2024-05-13

**Authors:** Nak-Hyun Kim, Kyungdo Han, Eunjeong Ji, Soyeon Ahn, Yunsang Choi, Seong Jin Choi, Song Mi Moon, Kyoung-Ho Song, Eu Suk Kim, Hong Bin Kim

**Affiliations:** 1 Department of Internal Medicine, Seoul National University Bundang Hospital, Seoul National University College of Medicine, Seongnam, Republic of Korea; 2 Department of Statistics and Actuarial Science, Soongsil University, Seoul, Republic of Korea; 3 Medical Research Collaborating Center, Seoul National University Bundang Hospital, Seongnam, Republic of Korea

## Abstract

**Objective:**

This study aimed to assess the actual burden of antibiotic use among end-of-life (EOL) patients in South Korea and to compare trends between cancer and non-cancer decedents.

**Design:**

Population-based mortality follow-back study.

**Setting:**

Data from the Korean National Health Insurance Database, covering the period from January1, 2006, to December 31, 2018, provided for research by the National Health Insurance Service (NHIS), were used.

**Participants:**

All decedents from 2006 to 2018 were included and categorized as cancer decedents or non-cancer decedents.

**Methods:**

Annual antibiotic consumption rates and prescription rates were calculated, and Poisson regression was used to estimate their trends.

**Results:**

Overall antibiotic consumption rates decreased slightly among decedents in their final month with a less pronounced annual decrease rate among cancer decedents compared to non-cancer decedents (0.4% vs 2.3% per year, *P* <.001). Over the study period, although narrow spectrum antibiotics were used less, utilization and prescription of broad-spectrum antibiotics steadily increased, and prescription rates were higher in cancer decedents compared to non-cancer controls. Specifically, carbapenem prescription rates increased from 5.6% to 18.5%, (RR 1.087, 95% CI 1.085–1.088, *P* <.001) in cancer decedents and from 2.9% to 13.2% (RR 1.115, 95% CI 1.113–1.116, *P* <.001) in non-cancer decedents.

**Conclusions:**

Our findings show that patients at the EOL, especially those with cancer, are increasingly and highly exposed to broad-spectrum antibiotics. Measures of antibiotic stewardship are required among this population.

## Introduction

Patients at the terminal stages of cancer typically grapple with various medical, psychological, and social challenges; given that this condition is incurable and ultimately leads to death,^1^ current clinical practice guidelines emphasize the early integration of palliative care into standard care to improve quality of life and alleviate psychological distress in these patients.^2,3^ Patients with advanced cancer are also highly vulnerable to infections, and frequently exhibit symptoms and signs that are hard to differentiate from those of infection.^4^ Even in terminally ill patients under palliative care, acute infections are generally considered treatable and rarely the reason for palliative care.^5^ Given the unpredictability of the residual life span and the perception of antibiotics as less burdensome compared to other potentially life-prolonging interventions,^6^ the use of antibiotics is highly prevalent during palliative care.^4,6^

The evidence supporting the use of antibiotics in palliative care settings is limited^3,7^; additionally, antibiotic exposure is known to be associated with potential adverse effects, including drug reactions, *Clostridioides difficile* infection, the acquisition of multidrug-resistance, increasing healthcare costs, and public health risks associated with antimicrobial resistance (AMR) (as patients receiving antibiotics during end-of-life (EOL) care may become reservoirs for resistant bacteria).^4,7,8^ Considering that cancer is the second leading cause of death in most Organization for Economic Cooperation and Development (OECD) countries and accounts for approximately one-fourth of all deaths,^9^ antibiotic use among patients with terminal cancer under palliative care is an important target for antimicrobial stewardship.

Although many studies report a significant prevalence of antibiotic use during EOL care,^4,6,8^ most of these studies are based on a small sample size rather than population-based data, and reports on antibiotic consumption in patients with cancer nearing the EOL using standard metrics are scarce. The aim of this study was to analyze the amounts of antibiotic consumption before death in patients with cancer, over the years 2006 to 2018, focusing on different time intervals (1 month, 6 months, and 1 year) during their final year of life by using population-based data from the Korean National Health Insurance Database (KNHID).

## Methods

### Data source

This study used data from the KNHID, covering the period from January 1, 2006, to December 31, 2018, provided for research by the National Health Insurance Service (NHIS), a mandatory universal public health insurance system which provides coverage for almost the entire Korean population and reimburses healthcare providers through a fee-for-service system.^10^ The KNHID data encompass inpatient and outpatient information and include demographic characteristics, diagnostic codes from the International Classification of Disease 10th revision (ICD-10), medical service utilization, prescription records, and death information for all enrolled Korean citizens. Because the KNHID data were collected for health insurance claims, the codes of tests and procedures performed are available, but their results are not included. As antibiotics can only be obtained via prescriptions in Korea, all antibiotic utilization data are recorded in the NHID.

### Study population

All decedents were included and followed-back for their final year of life and categorized as either cancer or non-cancer decedents. Cancer decedents were defined as those who used healthcare services associated with the ICD-10 codes for cancer (C00∼C97) and an active specific insurance code “V193,” which is representative of active status of malignancy. Code V193 is activated at the time of cancer diagnosis and is periodically updated for patients who are actively treated or followed-up for cancer. In successfully treated cancer patients, the code is deactivated 5 years after diagnosis. The cancer decedents were further classified according to the type of cancer. In patients with multiple types of cancers, only the primary diagnosis was considered. Decedents without an active diagnosis of cancer were used as the control group.

### Antibiotics and outcomes

The medication prescription data were mapped to the World Health Organization’s Anatomic Therapeutic Chemical Classification (ATC) system.^11^ We included systemic antibacterials (ATC classification J01) and classified the antibiotics according to the third level of classification, referring to the pharmacologic subgroup. For each antibiotic subgroup, antibiotic consumption rates and prescription rates were calculated. Antibiotic prescription rates were defined as the proportion of patients who received antibiotics among all decedents for inpatients and outpatients. Antibiotic consumption rates for each type of cancer were expressed as days of therapy (DOT) per 1,000 patient-days using annual inpatient data. DOT was estimated by counting each day a specific antibiotic was prescribed to a patient.^12^ Definitions for broad-spectrum antibiotics were adapted from the Centers for Disease Control and Prevention (CDC) National Healthcare Safety Network’s (NHSN) Antimicrobial Use and Resistance Module.^13^

### Statistical analysis

Data are presented as means ± standard deviations (SDs) and ranges for continuous variables and as numbers and percentages for categorical variables. Annual trends in antibiotic use were analyzed using Poisson regression models. Each model included an offset term for total number of deaths or total prescription days. Subgroup analyses were performed on cancer and non-cancer decedents by cancer type and period before death. The rate ratios (RR) and 95% CI were calculated, and a two-sided *P* value less than .05 was considered statistically significant. SAS version 9.4 (SAS Institute Inc., Cary, North Carolina, USA) and R version 4.1.1 (The R Foundation for Statistical Computing, Vienna, Austria) were used for statistical analysis.

### Ethics

As this study used public data which did not contain any identifying information, it was exempted from ethical review by the Seoul National University Bundang Hospital’s Institutional Review Board (X-2007-625-902).

## Results

### Demographic characteristics of the study population

From 2006 to 2018, the total study population comprised 3,437,670 decedents, with 965,943 (28.1%) having cancer and 2,471,727 (71.9%) without cancer. The average annual number of deaths was 264,436 ± 17,995 (range, 241,813–299,610), of these 28% (average, 74,303 ± 8,154; range, 58,517–85,372) of deaths were owing to cancer. The most prevalent cancers were lung cancer, followed by liver cancer and stomach cancer. Forty-six percent of non-cancer decedents died in their 80s, whereas cancer decedents died most frequently in their 70s and 60s (Table 1).

**Table 1.** Demographic characteristics of the study population

Year		2006	2007	2008	2009	2010	2011	2012	2013	2014	2015	2016	2017	2018	Total
Total Decedents		241,813	244,625	245,865	246,590	255,051	257,427	267,429	266,572	268,437	276,454	281,444	286,353	299,610	3,437,670
**Non-cancer Decedents**	N	183,296	181,091	179,163	177,321	182,616	184,249	192,660	189,928	189,694	196,148	198,984	202,339	214,238	2,471,727
	%	75.8	74.0	72.9	71.9	71.6	71.6	72.0	71.2	70.7	71.0	70.7	70.7	71.5	71.9
Age group (years)															
-17		2,485	2,441	2,323	2,260	2,069	2,019	1,885	1,774	1,804	1,552	1,529	1,437	1,345	24,923
18–29		3,493	4,001	3,963	3,978	3,757	3,419	2,966	2,770	2,625	2,558	2,526	2,449	2,464	40,969
30–39		5,596	5,718	5,602	5,981	5,666	5,386	5,040	4,772	4,586	4,103	3,950	3,748	3,915	64,063
40–49		12,896	12,415	12,250	12,105	11,880	11,084	10,551	10,372	9,740	9,338	8,900	8,548	8,553	138,632
50–59		16,386	15,878	16,099	16,521	17,265	17,749	17,656	17,732	17,526	16,954	16,719	16,204	16,717	219,406
60–69		25,776	24,227	22,810	21,833	21,444	20,458	19,545	19,002	19,010	19,781	20,469	19,834	21,574	275,763
70–79		46,493	45,364	44,187	43,278	44,658	44,913	47,838	46,307	44,417	44,331	43,077	41,783	42,513	579,159
80-		70,171	71,047	71,929	71,365	75,877	79,221	87,179	87,199	89,986	97,531	101,814	108,336	117,157	1,128,812
**Cancer Decedents**	N	58,517	63,534	66,702	69,269	72,435	73,178	74,769	76,644	78,743	80,306	82,460	84,014	85,372	965,943
	%	24.2	26.0	27.1	28.1	28.4	28.4	28.0	28.8	29.3	29.0	29.3	29.3	28.5	28.1
Age group (years)															
-17		285	334	256	260	254	258	250	217	205	186	197	180	168	3,050
18–29		432	409	470	423	389	422	370	356	317	380	337	311	318	4,934
30–39		1,460	1,524	1,431	1,413	1,351	1,213	1,178	1,178	1,111	1,135	1,043	997	974	16,008
40–49		4,999	5,035	5,050	4,953	4,759	4,494	4,380	4,354	4,234	4,046	3,937	3,663	3,441	57,345
50–59		9,713	10,003	10,313	10,649	10,888	11,322	11,396	11,512	11,687	11,268	11,176	10,603	10,275	140,805
60–69		16,159	16,990	17,085	17,015	17,250	16,517	15,820	16,086	16,167	16,799	17,258	17,441	17,408	217,995
70–79		17,875	20,104	21,528	22,721	23,958	24,519	25,678	26,226	26,332	26,143	26,045	26,408	26,255	313,792
80-		7,594	9,135	10,569	11,835	13,586	14,433	15,697	16,715	18,690	20,349	22,467	24,411	26,533	212,014
Type of Cancer															
Stomach		6,453	6,988	7,469	7,781	7,771	7,728	7,428	7,546	7,358	7,475	7,417	7,275	7,289	95,978
Colorectal		3,405	4,146	4,564	4,910	5,479	5,624	5,973	5,985	6,230	6,418	6,734	6,995	7,235	73,698
Liver		8,659	9,064	9,364	9,725	9,567	9,595	10,056	10,053	10,385	10,234	10,191	10,122	10,180	127,195
Biliary		1,982	2,183	2,348	2,443	2,506	2,604	2,646	2,642	2,832	3,068	3,184	3,460	3,573	35,471
Pancreatic		2,129	2,217	2,510	2,655	2,818	2,925	3,201	3,358	3,605	3,993	4,220	4,302	4,577	42,510
Lung		8,236	9,019	9,991	10,020	10,712	11,026	11,787	12,305	12,572	12,900	13,411	13,409	13,585	148,973
Breast		722	835	932	1,098	1,060	1,186	1,159	1,327	1,413	1,572	1,670	1,688	1,743	16,405
Thyroid		246	350	364	408	431	528	495	518	545	578	576	546	543	6,128
Hematologic		2,610	2,957	3,097	3,273	3,497	3,421	3,743	3,842	3,974	4,315	4,713	4,844	5,028	49,314
Others		24,075	25,775	26,063	26,956	28,594	28,541	28,281	29,068	29,829	29,753	30,344	31,373	31,619	370,271

### Trends of antibiotic use in the last month before death

During the study period, inpatient antibiotic consumption rates during the last month of life decreased for both cancer decedents and non-cancer controls; for cancer decedents, antibiotic consumption decreased by 4.8% (39.8 DOT/1,000pt-days) from 830.9 DOT/1,000pt-days to 791.0 DOT/1,000pt-days, with an estimated decrease in antibiotic consumption rates of 0.4% (RR 0.996, 95%CI, 0.996–0.996) per year. For non-cancer decedents, antibiotic consumption decreased by 27.1% (282.8 DOT/1,000pt-days) from 1041.9 DOT/1,000pt-days to 759.1 DOT/1,000pt-days, with an estimated decrease of 2.3% (RR 0.977, 95%CI, 0.977–0.977) per year (Table 2, Supplementary Figure 1). Comparably, cumulative antibiotic prescription rates during the last month of life, while being higher in cancer decedents compared to non-cancer decedents, continuously increased over the study years (Table 3, Supplementary Figure 2).

**Table 2.** Antibiotic consumption rates (days-of-therapy/1,000 patient-days) and their temporal trends in cancer and non-cancer decedents during their last month of life, 2006–2018

Year		2006	2007	2008	2009	2010	2011	2012	2013	2014	2015	2016	2017	2018	RR	Lower CI	Upper CI	*P* value
Overall	Non-Cancer	1041.9	1007.9	857.2	886.4	853.5	812.9	808.2	783.0	776.2	769.5	754.9	750.1	759.1	0.977	0.977	0.977	.0000
	Cancer	830.9	836.4	794.8	807.9	791.7	766.7	772.3	764.4	773.4	769.4	771.2	782.8	791.0	0.996	0.996	0.996	.0000
1st generation cephalosporins	Non-Cancer	80.0	76.4	52.9	46.1	43.0	35.0	30.8	26.3	23.1	19.0	17.5	16.1	14.4	0.861	0.860	0.861	.0000
	Cancer	31.8	32.1	25.0	23.6	21.4	18.0	15.5	13.5	12.0	10.9	11.8	11.9	10.6	0.902	0.901	0.903	.0000
2nd generation cephalosporins	Non-Cancer	95.6	88.0	58.5	49.9	40.5	35.4	29.6	25.0	23.1	21.7	18.6	16.9	15.0	0.851	0.850	0.851	.0000
	Cancer	66.8	58.2	43.8	38.7	29.6	25.8	21.6	19.6	19.7	18.1	15.1	14.0	11.6	0.865	0.864	0.866	.0000
3rd generation cephalosporins	Non-Cancer	243.3	233.3	208.8	216.9	209.1	191.1	185.4	170.4	160.3	152.3	143.0	140.3	136.4	0.950	0.950	0.951	.0000
	Cancer	253.4	252.4	234.2	229.4	221.3	208.2	196.4	188.0	184.3	178.8	176.1	174.5	171.3	0.965	0.965	0.965	.0000
4th generation cephalosporins	Non-Cancer	5.8	6.8	7.1	9.1	9.5	10.7	11.5	11.5	12.7	14.1	14.2	15.2	14.2	1.071	1.070	1.072	.0000
	Cancer	11.6	13.9	15.7	19.1	21.1	21.7	21.3	22.6	22.7	23.9	23.1	24.4	21.8	1.039	1.038	1.040	.0000
Aminoglycosides	Non-Cancer	204.9	178.4	135.3	127.0	108.4	86.1	74.5	63.2	55.7	48.6	44.3	42.2	41.7	0.864	0.864	0.865	.0000
	Cancer	129.4	114.6	89.6	77.0	63.4	46.5	39.6	32.4	29.0	23.4	21.0	20.2	19.9	0.836	0.836	0.837	.0000
β-lactam/ β-lactamase inh combinations	Non-Cancer	65.1	68.6	66.2	74.2	80.0	84.1	94.5	102.2	107.4	116.5	120.9	125.2	140.3	1.070	1.069	1.070	.0000
	Cancer	56.6	64.5	72.0	81.9	88.3	98.0	111.5	122.6	131.1	139.0	146.3	150.3	165.3	1.086	1.085	1.086	.0000
Carbapenems	Non-Cancer	42.9	48.2	50.3	62.5	64.8	70.3	77.7	83.6	92.5	96.7	100.6	103.6	107.2	1.075	1.074	1.075	.0000
	Cancer	40.0	44.7	50.4	60.8	65.2	67.3	76.4	83.5	88.9	90.7	91.6	98.3	99.8	1.070	1.070	1.071	.0000
Fluoroquinolones	Non-Cancer	122.3	128.3	116.2	125.8	123.6	127.4	128.5	128.1	130.3	132.3	132.0	128.9	129.3	1.006	1.006	1.006	.0000
	Cancer	99.4	106.1	112.9	121.3	121.7	125.4	130.7	126.7	128.9	127.6	128.8	128.2	129.3	1.015	1.015	1.016	.0000
Glycopeptides	Non-Cancer	29.8	33.2	36.3	41.0	43.2	45.8	48.9	50.7	51.3	51.1	50.5	50.1	51.3	1.036	1.035	1.036	.0000
	Cancer	22.7	30.6	36.0	39.8	43.2	44.9	48.6	48.1	49.1	49.0	48.8	52.4	51.9	1.044	1.043	1.045	.0000
Macrolides	Non-Cancer	28.7	27.0	23.5	22.4	21.2	20.3	20.6	16.9	15.7	14.7	12.8	11.7	11.4	0.925	0.925	0.926	.0000
	Cancer	19.6	20.1	17.1	16.9	15.9	13.8	13.7	11.7	11.5	10.4	9.4	7.8	6.5	0.917	0.916	0.919	.0000
Oxazolinediones	Non-Cancer	0.0	0.4	0.7	0.9	1.3	1.3	1.7	1.4	1.5	1.6	1.5	1.8	1.7	1.092	1.088	1.096	.0000
	Cancer	0.0	0.5	1.0	1.0	1.2	1.5	1.6	1.7	2.0	1.8	2.1	2.3	2.7	1.127	1.122	1.132	.0000
Penicillins	Non-Cancer	10.1	9.4	5.5	5.2	3.9	3.5	2.9	3.0	3.0	2.8	2.9	2.9	2.7	0.894	0.892	0.896	.0000
	Cancer	4.9	4.7	3.2	3.1	2.7	2.6	2.4	2.1	2.1	2.4	2.4	2.3	2.2	0.940	0.937	0.943	.0000
Polymyxins	Non-Cancer	0.0	0.9	2.0	1.6	3.5	5.1	7.1	7.8	9.5	9.7	10.6	10.4	10.4	1.176	1.174	1.178	.0000
	Cancer	0.0	0.4	1.5	0.9	2.3	2.8	3.7	4.2	4.5	4.4	4.7	4.7	5.1	1.151	1.147	1.154	.0000
Others	Non-Cancer	113.4	108.9	94.0	103.6	101.5	96.8	94.5	92.9	90.2	88.5	85.4	84.9	83.1				
	Cancer	94.7	93.5	92.3	94.4	94.2	90.2	89.2	87.8	87.6	89.2	90.0	91.7	92.9				

RR: Rate ratio; CI: Confidence interval; inh: inhibitor.

**Table 3.** Antibiotic prescription rates (%) and their temporal trends in cancer and non-cancer decedents during their last month of life, 2006-2018

Year		2006	2007	2008	2009	2010	2011	2012	2013	2014	2015	2016	2017	2018	RR	Lower CI	Upper CI	*P* value
1st generation cephalosporins	Non-Cancer	6.8	7.5	6.7	6.3	6.3	5.7	5.5	5.1	4.9	4.4	4.3	4.2	3.8	0.948	0.947	0.949	.0000
	Cancer	5.2	5.6	5.1	5.0	4.9	4.5	4.3	3.8	3.6	3.5	3.6	3.6	3.4	0.956	0.954	0.959	.0000
2nd generation cephalosporins	Non-Cancer	9.1	9.6	8.6	8.4	7.9	7.5	7.4	6.9	6.9	6.8	6.4	6.3	6.0	0.963	0.962	0.964	.0000
	Cancer	10.0	9.7	8.6	8.0	7.0	6.3	5.9	5.6	5.6	5.4	4.8	4.8	4.3	0.932	0.930	0.934	.0000
3rd generation cephalosporins	Non-Cancer	17.3	19.8	20.5	21.9	22.7	22.3	23.0	22.3	22.3	22.3	22.0	22.4	22.0	1.012	1.011	1.012	.0000
	Cancer	31.6	33.7	34.2	34.7	35.2	34.0	33.2	32.4	32.0	31.9	32.1	33.0	33.2	0.997	0.996	0.997	.0000
4th generation cephalosporins	Non-Cancer	0.5	0.7	0.8	1.1	1.2	1.4	1.5	1.5	1.8	2.0	2.0	2.3	2.1	1.109	1.106	1.112	.0000
	Cancer	2.0	2.6	3.2	3.7	4.2	4.5	4.5	4.6	4.7	4.8	4.9	5.3	4.9	1.056	1.053	1.059	.0000
Aminoglycosides	Non-Cancer	15.6	16.1	14.4	13.9	13.0	11.3	10.3	9.1	8.5	7.8	7.3	7.2	6.8	0.923	0.923	0.924	.0000
	Cancer	19.3	18.4	16.2	14.6	12.9	10.1	8.7	7.3	6.8	5.8	5.2	5.0	4.9	0.876	0.874	0.877	.0000
β-lactam/lactamase inh combinations	Non-Cancer	6.0	7.4	8.5	9.5	10.8	11.7	13.4	14.4	15.8	17.0	18.1	19.0	20.8	1.097	1.096	1.098	.0000
	Cancer	8.9	10.5	13.1	14.8	16.9	18.6	21.2	22.7	23.9	25.5	27.0	28.6	31.1	1.094	1.092	1.095	.0000
Carbapenems	Non-Cancer	2.9	3.9	4.7	5.8	6.6	7.4	8.5	9.4	10.5	11.3	12.0	12.8	13.2	1.115	1.113	1.116	.0000
	Cancer	5.6	6.7	8.3	9.8	11.4	11.8	13.4	14.4	15.4	15.8	16.4	17.8	18.5	1.087	1.085	1.088	.0000
Fluoroquinolones	Non-Cancer	10.2	12.0	12.7	14.1	15.0	15.8	16.7	16.9	17.6	18.0	18.3	18.5	18.7	1.044	1.043	1.045	.0000
	Cancer	14.8	16.6	18.6	19.7	21.2	22.1	23.3	22.9	23.4	23.4	23.9	24.3	24.7	1.034	1.032	1.035	.0000
Glycopeptides	Non-Cancer	2.2	2.9	3.6	4.3	5.0	5.5	6.2	6.6	6.8	7.1	7.2	7.4	7.6	1.085	1.083	1.087	.0000
	Cancer	3.4	4.9	6.4	7.4	8.7	9.3	10.0	10.0	10.3	10.4	10.6	11.6	11.8	1.070	1.068	1.072	.0000
Macrolides	Non-Cancer	3.0	3.3	3.6	3.7	3.8	3.8	4.1	3.8	3.8	3.9	3.6	3.5	3.4	1.006	1.004	1.007	.0000
	Cancer	3.8	4.3	4.2	4.2	4.3	3.9	4.0	3.6	3.6	3.4	3.4	3.0	2.7	0.969	0.966	0.972	.0000
Oxazolinediones	Non-Cancer	0.0	0.0	0.1	0.1	0.1	0.1	0.2	0.2	0.2	0.2	0.2	0.2	0.2	1.138	1.128	1.150	.0000
	Cancer	0.0	0.1	0.1	0.2	0.2	0.3	0.3	0.3	0.4	0.3	0.4	0.4	0.5	1.145	1.133	1.158	.0000
Penicillins	Non-Cancer	1.5	1.6	1.3	1.2	1.1	1.0	1.0	0.9	0.9	0.9	0.9	0.8	0.8	0.936	0.934	0.938	.0000
	Cancer	1.2	1.3	1.0	1.0	0.9	0.9	0.8	0.8	0.7	0.8	0.7	0.8	0.7	0.943	0.940	0.947	.0000
Polymyxins	Non-Cancer	–	0.1	0.2	0.2	0.4	0.6	0.8	0.9	1.1	1.2	1.3	1.3	1.3	1.210	1.204	1.215	.0000
	Cancer	–	0.1	0.3	0.2	0.5	0.6	0.8	0.8	0.9	0.9	0.9	1.0	1.0	1.160	1.151	1.169	.0000
Others	Non-Cancer	10.9	12.0	12.1	13.2	13.6	13.3	13.5	13.3	13.3	13.2	13.1	13.3	13.1	1.392	1.346	1.442	.0000
	Cancer	16.6	17.5	18.4	18.9	19.6	18.7	18.6	18.1	17.9	17.8	18.0	18.7	18.7	1.340	1.277	1.409	.0000
Cumulative prescription rates*	Non-Cancer	86.0	96.9	97.8	104.0	107.4	107.4	112.1	111.3	114.3	116.1	116.7	119.1	120.1				
	Cancer	122.4	131.9	137.6	142.2	147.9	145.6	148.9	147.3	149.1	149.7	152.0	157.9	160.3				

RR: Rate ratio; CI: Confidence interval; inh: inhibitor.

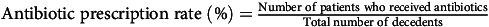
 .

* Cumulative Prescription Rate: The sum of the prescription rates of all antibiotic subgroups during the study year.

Antibiotic consumption rates and prescription rates varied between cancer decedents and non-cancer controls, and time trends were diverse across different subclasses of antibiotics (Tables 2 and 3, Supplementary Figures 3 and 4). The most frequently used antibiotic subgroups in 2006 were the 3^rd^ generation cephalosporins, fluoroquinolones, and aminoglycosides, whereas in 2018, the 3^rd^ generation cephalosporins, beta-lactam/beta-lactamase inhibitor combinations and fluoroquinolones were most frequently used (Tables 2 and 3). In 2018, 33.2% of cancer decedents received 3^rd^ generation cephalosporins during their last month of life, compared to 22.0% of non-cancer decedents. The proportion of cancer decedents and non-cancer decedents that received beta-lactam/beta-lactamase inhibitor combinations were 31.1% and 20.8%, respectively (Table 3).

Annual trends of consumption rates for each antibiotic subgroup were similar for cancer decedents and non-cancer controls: decreasing trends were observed for 1^st^, 2^nd^, and 3^rd^ generation cephalosporins, penicillins, aminoglycosides, and macrolides, whereas increasing trends were seen for 4^th^ generation cephalosporins, beta-lactam/beta-lactamase inhibitor combinations, fluoroquinolones, carbapenems, glycopeptides, oxazolidinediones, and polymyxins (Table 2 and Supplementary Figure 3). Antibiotic prescription rates over the study period changed similarly in cancer decedents and non-cancer decedents for most antibiotic subclasses with the exception of 3^rd^ generation cephalosporins and macrolides; prescription rates of 3^rd^ generation cephalosporins on average decreased by 0.3% (RR 0.997, 95% CI 0.996–0.997) for each study year in cancer decedents and increased by 1.2% (RR 1.012, 95% CI 1.011–1.012) for each study year in non-cancer decedents, and prescription rates of macrolides on average decreased by 3.1% (RR 0.969, 95% CI 0.966–0.972) per study year in cancer decedents and increased by 0.6% (RR 1.006, 95% CI 1.004–1.007) per study year in non-cancer decedents (Table 3 and Supplementary Figure 4). In all other antibiotic subgroups (excluding 3^rd^ generation cephalosporins and macrolides), annual trends of prescription rates were comparable to those of consumption rates. The most pronounced increase in prescription rates was seen in oxazolidinediones, and polymyxins. Among cancer decedents, oxazolidinedione prescription rates on average increased by 14.5% (RR 1.145, 95% CI 1.133–1.158) per study year and polymyxin prescription rates on average increased by 16.0% (RR 1.160, 95% CI 1.151–1.169) per study year. For non-cancer decedents, the corresponding increase in prescription rates were 13.8% (RR 1.138, 95% CI 1.128–1.150) per study year for oxazolidinediones and 21% (RR 1.210, 95% CI 1.204–1.215) per study year for polymyxins (Table 3 and Supplementary Figure 4).

**Figure 1.**
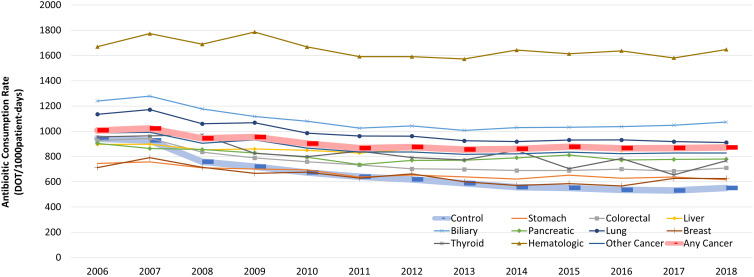
Overall antibiotic consumption rates (days-of-therapy/1,000pt-days) among cancer decedents according to underlying cancer type during the last month of life.

Patterns of annual antibiotic consumption rates were grossly similar across different cancer types, however patients with hematological malignancies showed significantly higher utilization rates by 1.7-fold compared to overall cancer decedents (Figure 1) and used significantly larger amounts of carbapenems, glycopeptides, oxazolidinediones, polymyxins, and 4^th^ generation cephalosporins in their last month of life. Likewise, beta-lactam/beta-lactamase inhibitor combinations, fluoroquinolones, and macrolides were used in greater amounts among patients with hematological malignancies and lung cancer during their last month of life (Supplementary Figure 5A). Prescription rates of antibiotic subgroups for different types of cancer were grossly similar to those of antibiotic consumption rates (Supplementary Figure 5B).

### Trends of antibiotic use at different time intervals in the last year before death

In both cancer decedents and non-cancer decedents, prescription rates of all antibiotic subgroups showed a uniform decreasing trend according to the timespan before death, with rates being highest in the 1 year preceding death, followed by 6 months before death, and then 1 month before death. However, for beta-lactam/beta-lactamase inhibitor combinations, carbapenems, glycopeptides, and polymyxins, prescription rates during 1 month before death exceeded 50% of the rates during 1 year before death (Supplementary Figure 6).

**Figure 2.**
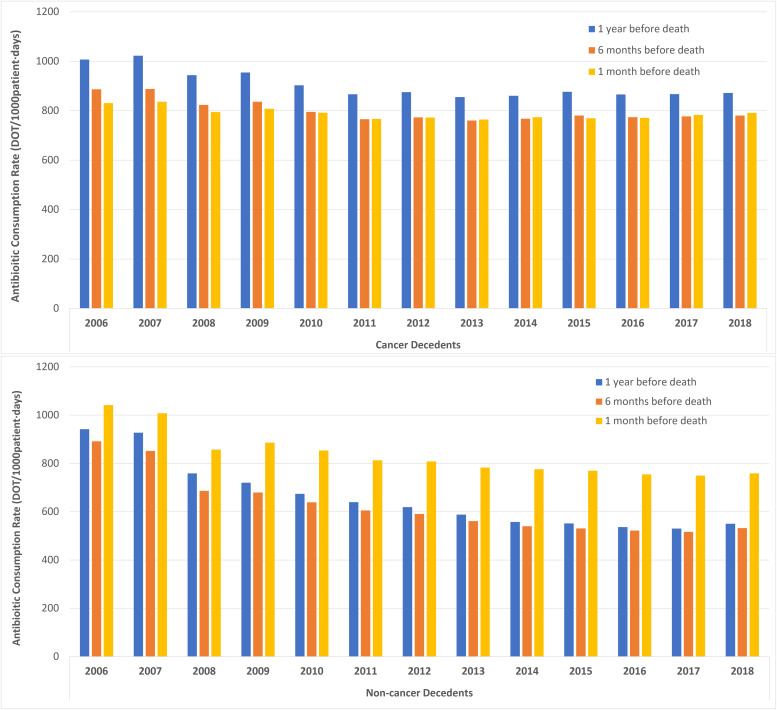
Overall antibiotic consumption rates (days-of-therapy/1,000pt-days) in cancer and non-cancer decedents according to timespan preceding death (1 year, 6 months, and 1 month before death).

Overall antibiotic consumption rates in cancer decedents and non-cancer decedents steadily decreased during the study period regardless of the time frames preceding death (specifically, 1 year, 6 months, and 1 month before death). Overall antibiotic consumption rates in cancer decedents were greatest over the last 1 year prior to death with an average annual decrease of 1.2% (RR 0.988, 95%CI, 0.988–0.988), and least in their last month which on average decreased by 0.4% (RR 0.996, 95%CI, 0.996–0.996) per study year. In contrast, overall antibiotic consumption rates in non-cancer decedents were greatest during their last month of life which on average decreased by 2.3% (RR 0.977, 95%CI, 0.977–0.977) per year, and least over the last 6 months prior to death which decreased by 4.1% (RR 0.959, 95%CI, 0.959–0.959) per year during the study period (Figure 2 and Supplementary Table 1). In contrast to the decreasing trends of overall antibiotic consumption rates in cancer decedents at different timespans before death, beta-lactam/beta-lactamase inhibitor combinations, carbapenems, and polymyxins were used most during the last month of life (Figure 2, Supplementary Figure 7, and Supplementary Table 1).

## Discussion

Antibiotics are commonly used at the EOL, although their benefit in terminally ill patients is debatable.^4,6^ This population-based longitudinal cohort study, based on Korean national claims data from 2006 to 2018, examines antibiotic use among EOL patients and compares trends between cancer and non-cancer decedents. Key findings include a slight decrease in overall antibiotic consumption rates in the last month before death with a less pronounced annual decrease rate among cancer decedents compared to non-cancer decedents (0.4% vs 2.3% per year, respectively, *P* <.001). Broad-spectrum antibiotics and antibiotics for resistant bacteria increased while narrow spectrum antibiotics decreased, with overall higher prescription rates among cancer decedents. The continuously increasing annual cumulative antibiotic prescription rates (Table 3, Supplementary Figure 2) show that increasing numbers of patients received treatment with more than one antibiotic in their last month. Our findings suggest that patients at EOL, particularly those with cancer, are increasingly and heavily exposed to broad-spectrum antibiotics, which, although likely a consequence of increased AMR over time, poses a great threat for further AMR emergence and spread. There is a need to critically assess the tangible benefits of antibiotic use in EOL care and reconsider the perceptions of its noninvasive nature.

Previous studies on antibiotic use near the EOL show similar findings. A systematic review and meta-analysis by Marra et al on the burden of EOL antibiotics, based on 72 studies with various populations found that over half of EOL patients were exposed to antibiotics (cancer patients, range 26.8%–100%).^4^ A multicenter retrospective cohort study conducted by Wi et al in 2018 with 13 tertiary hospitals in South Korea showed that 88.9% of patients received a median of 2 antibiotics during the last 14 days of life and 83.8% received antibiotics until death.^14^ Another retrospective study from a tertiary medical center during 2019–2021 reported that 96% of cancer patients received antibiotics, 76.5% received antibiotic combinations during EOL care, and 81.1% received antibiotics until death.^15^

Regarding the factors of antibiotic use in patients nearing the EOL, literature reviews by Fairweather et al^16^ and Macedo et al^17^ reveal inconsistencies and challenges in antibiotic decision-making for EOL patients, driven by ethical and legal uncertainties. The unpredictability of death, even during EOL care, may raise concerns about the potential for reversing infectious diseases in terminally ill patients, and the complex nature of these patients’ conditions often makes the diagnosis of infections difficult, if not impossible.^5,17^ In these circumstances, physicians may hesitate suspending antibiotic treatment for a potentially reversible infectious disease, fearing it could shorten the patients’ life.^17,18^ In the present study, antibiotic use in patients with hematological malignancies was observed to be especially extensive, and antibiotic utilization rates of the last month before death were comparable with that of the last 6 months and the last 1 year, indicating heavy antibiotic use until near death, which may be explained on the aforementioned grounds.

As the value of high-quality EOL care gains more recognition, there is increasing emphasis on comfort-care and early integration of palliative care.^2^ In Korea, the enactment of the Life-Sustaining Treatment (LST) Decision Act in February 2018 aimed to honor the patient’s self-determination regarding EOL care processes.^19^ Despite this, studies show continued antibiotic use after transitioning to comfort care,^15,20–23^ and even in a study that suggested active palliative care consultations might reduce antibiotic use in patients with terminal cancer, 73.5% of these patients remained on antibiotics.^22^ Interestingly, Marra et al reported that entry into hospice and palliative care were associated with an increase in antibiotic prescriptions,^5^ possibly reflecting the perception that antibiotics are less invasive than other LSTs.^6,23^ However, although some patients might benefit from with antibiotic treatment and experience symptomatic improvement,^20,24,25^ ethical concerns that antibiotic treatment could prolong the dying process and increase discomfort, are also raised.^6,17,18^

Currently, antibiotics, unlike mechanical ventilator support or hemodialysis, are not classified as LSTs,^17,19^ and good practice guidelines on antimicrobial use in palliative care or EOL care patients have not been established. However, antimicrobial stewardship guidelines state that for terminally ill patients, support by antimicrobial stewardship programs should be provided to clinical care providers related to antimicrobial treatment, and that antibiotic therapy should be viewed as aggressive care in the EOL setting.^12^ The absence of antibiotic treatment guidelines during EOL periods may contribute to excessive and indiscriminate antibiotic use,^17,18,26^ underscoring the need for discussions on antibiotic therapy during earlier stages of advanced care planning to reduce antibiotic misuse.^6,12^ A recently published state-of-the-art review highlighted the complexity of antimicrobial use at the EOL and proposed ongoing collaborative discussions between infectious diseases physicians and palliative care teams starting early in the disease course.^27^ It suggested employing a structured framework (REMAP), and considering a time-limited trial as an approach to meet the needs of patients and caregivers in the setting of uncertain prognoses.^27^

Potential strengths of our study are as follows. First, as we used national claims data, our study population is representative of all decedents in Korea during the study period, and despite the potential of discrepancies between the actual disease and the diagnosis entered, decedents with an active status of cancer could be distinguished specifically by means of the insurance code V193. Second, as antibiotics in Korea are only available by prescription, most data on antibiotic utilization could be obtained almost completely.

Despite the representativeness of our data, some potential limitations have to be taken into account. First, as claims data lack specific clinical and laboratory data including microbiological test results or clinical severity, it was not possible to assess the reason for antibiotic prescription or to determine the appropriateness of the prescribed antibiotics. Second, the lack of information regarding prior LST decisions prevented an assessment of their potential effect on antibiotic utilization. Third, details about the patients’ comorbid conditions were not available, and it also was difficult to assess the direct cause of death. While our study included all decedents with or without cancer during the study period, we acknowledge that the non-cancer group comprises individuals with various conditions, including those leading to acute mortality. This diversity accounts for a dissimilarity between the cancer and non-cancer control groups, potentially influencing the comparison of antibiotic usage between them. Fourth, antibiotic consumption rates were calculated for inpatients only. Therefore, it is possible that the consumption rates might have been underestimated for antibiotic subclasses available as oral agents and that can be prescribed at the outpatient clinic. However, as >90% of cancer decedents and >75% of all decedents die in hospitals in Korea,^28^ we believe that for the last month before death, the effect of this bias may be mitigated. Additionally, our study period unintentionally ended in 2018 due to the availability of data at the time of data extraction. The absence of data post-2018, including the COVID-19 pandemic period, warrants caution in interpreting findings during this specific time frame. Finally, as the analysis was conducted among antibacterials at the pharmacological subgroup level, issues related to antifungal stewardship which are also of importance were not addressed, and a more detailed analysis including medical costs from antibiotic use was not performed.

In conclusion, broad-spectrum antibiotics are highly and increasingly used during the EOL in Korea, especially among patients with cancer. Our findings underscore the importance of a more deliberate assessment to ascertain the utility of antibiotic usage in patients nearing the EOL. Early collaborative discussions concerning antibiotic administration should be integrated into the advanced care planning process.

## Supporting information

Kim et al. supplementary material Kim et al. supplementary material

## Data Availability

The datasets generated and analyzed during the current study are available from the corresponding author on reasonable request.

## Supplementary material

For supplementary material accompanying this paper visit http://doi.org/10.1017/ash.2024.75. click here to view supplementary material
