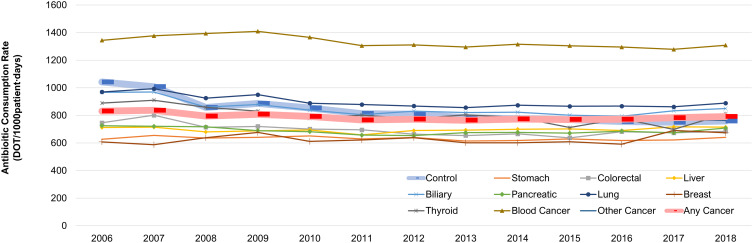# Trends of antibiotic use at the end-of-life of cancer and non-cancer decedents: a nationwide population-based longitudinal study (2006–2018) – CORRIGENDUM

**DOI:** 10.1017/ash.2025.10227

**Published:** 2025-11-10

**Authors:** Nak-Hyun Kim, Kyungdo Han, Eunjeong Ji, Soyeon Ahn, Yunsang Choi, Seong Jin Choi, Song Mi Moon, Kyoung-Ho Song, Eu Suk Kim, Hong Bin Kim

In this article,^1^ Figure 1 depicts antibiotic consumption rates from the last year of life, whereas the article’s intended and primary analysis focuses on data from the last month of life. The data analysis and conclusions presented in the text of the article are based on the correct data (antibiotic consumption rates from the last month of life), but the published figure does not accurately reflect this. A corrected version of Figure 1 is included in this corrigendum. The new figure accurately reflects the data from the last month of life, as discussed in the article. The authors apologize for the error.

**Figure:**