# Supplementary material for: Trends of antibiotic use at the end-of-life of cancer and non-cancer decedents: a nationwide population-based longitudinal study (2006–2018)
Source: Antimicrob Steward Healthc Epidemiol. 2024 May 13;4(1):e83. doi: 10.1017/ash.2024.75 (PMC11094383; doi:10.1017/ash.2024.75)
Supplement: Kim et al. supplementary material [file S2732494X24000755sup001.docx]

**SUPPLEMENTARY INFORMATION**

**LIST OF CONTENTS**

Supplementary Figure 1. Overall antibiotic consumption rates (days-of-therapy/1000pt-days) in cancer and non-cancer decedents during the last month of life

Supplementary Figure **2**. Cumulative prescription rates of antibiotic subclasses among cancer decedents and non-cancer decedents during the last month of life

Supplementary Figure 3. Consumption rates of antibiotic subclasses (days-of-therapy/1000pt-days) in cancer and non-cancer decedents during the last month of life

Supplementary Figure 4. Inpatient and outpatient prescription rates of antibiotic subclasses (%) in cancer and non-cancer decedents during the last month of life

Supplementary Figure 5.

A. Consumption rates of antibiotic subclasses (days-of-therapy/1000pt-days) among cancer decedents grouped by underlying cancer type compared to non-cancer decedents

B. Prescription rates of antibiotic subclasses (%) among cancer decedents grouped by underlying cancer type compared to non-cancer decedents

Supplementary Figure 6. Prescription rates of antibiotic subclasses in cancer and non-cancer decedents according to timespan preceding death (1 year, 6 months, and 1 month before death)

Supplementary Figure 7. Consumption rates of antibiotic subclasses in cancer and non-cancer decedents according to timespan preceding death (1 year, 6 months, and 1 month before death)

**Supplementary Table 1.** Temporal trends of antibiotic consumption rates in cancer and non-cancer decedents at different time intervals before death, 2006-2018

**Supplementary Figure 1.** Overall antibiotic consumption rates (days-of-therapy/1000pt-days) in cancer and non-cancer decedents during the last month of life


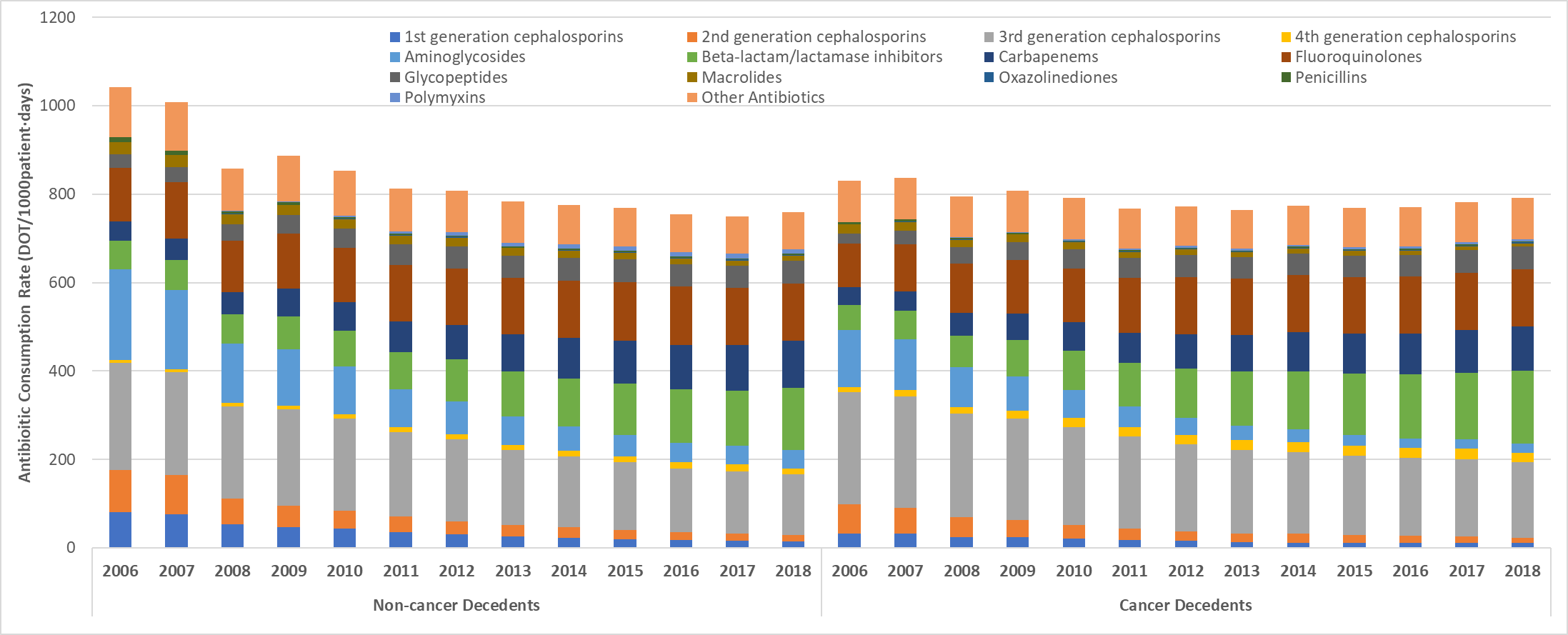


**Supplementary Figure 2**. Cumulative prescription rates of antibiotic subclasses among cancer decedents and non-cancer decedents during the last month of life


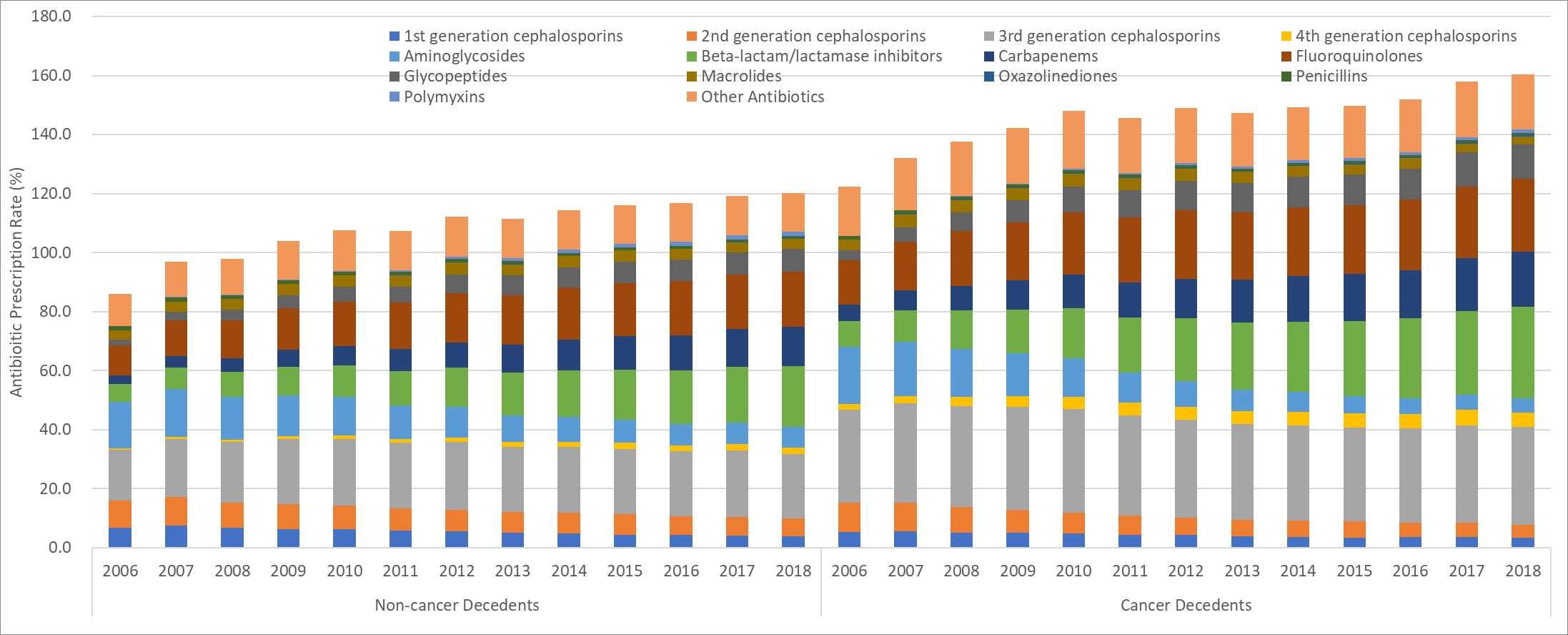


**Supplementary Figure 3**. Consumption rates of antibiotic subclasses (days-of-therapy/1000pt-days) in cancer and non-cancer decedents during the last month of life


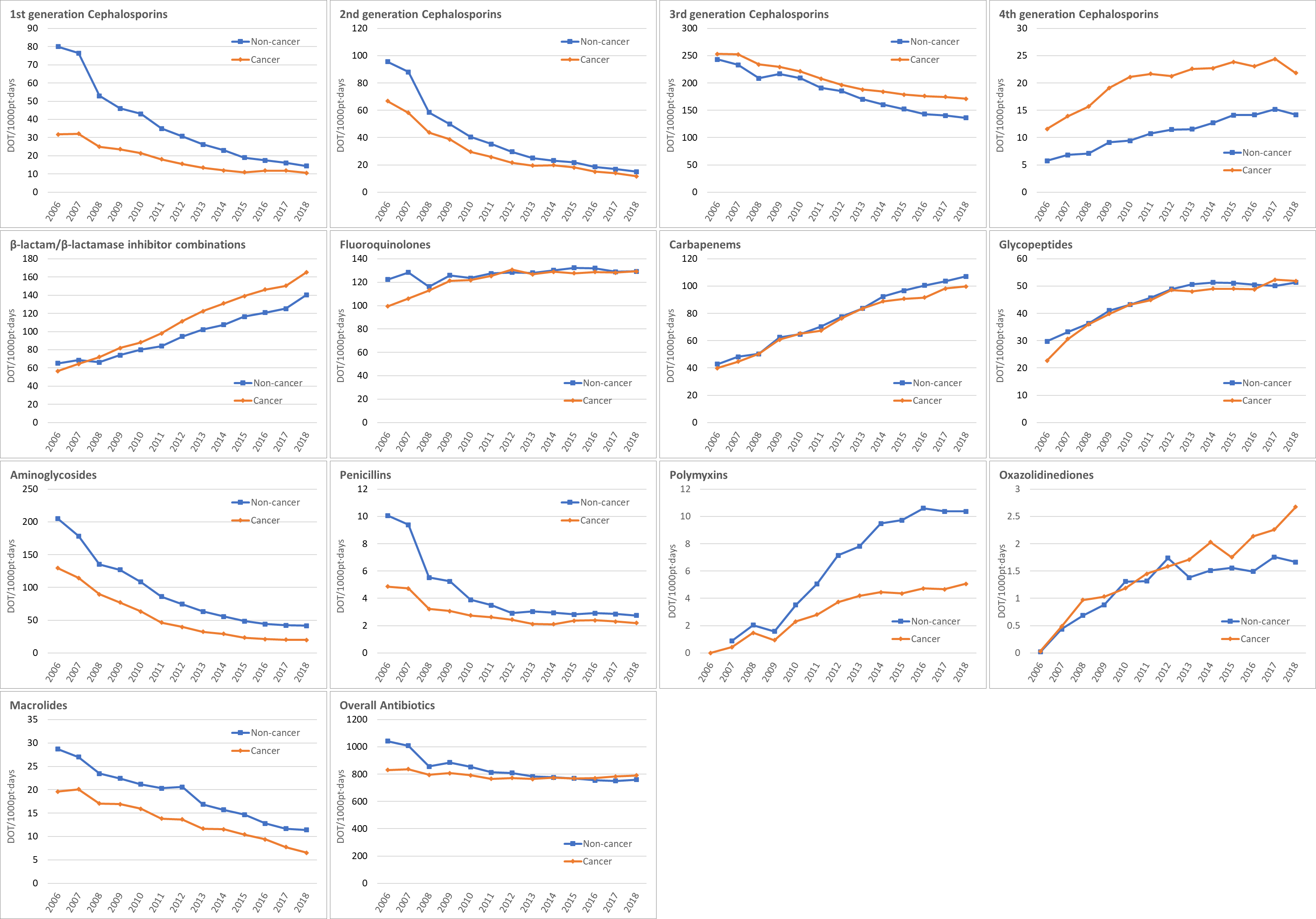


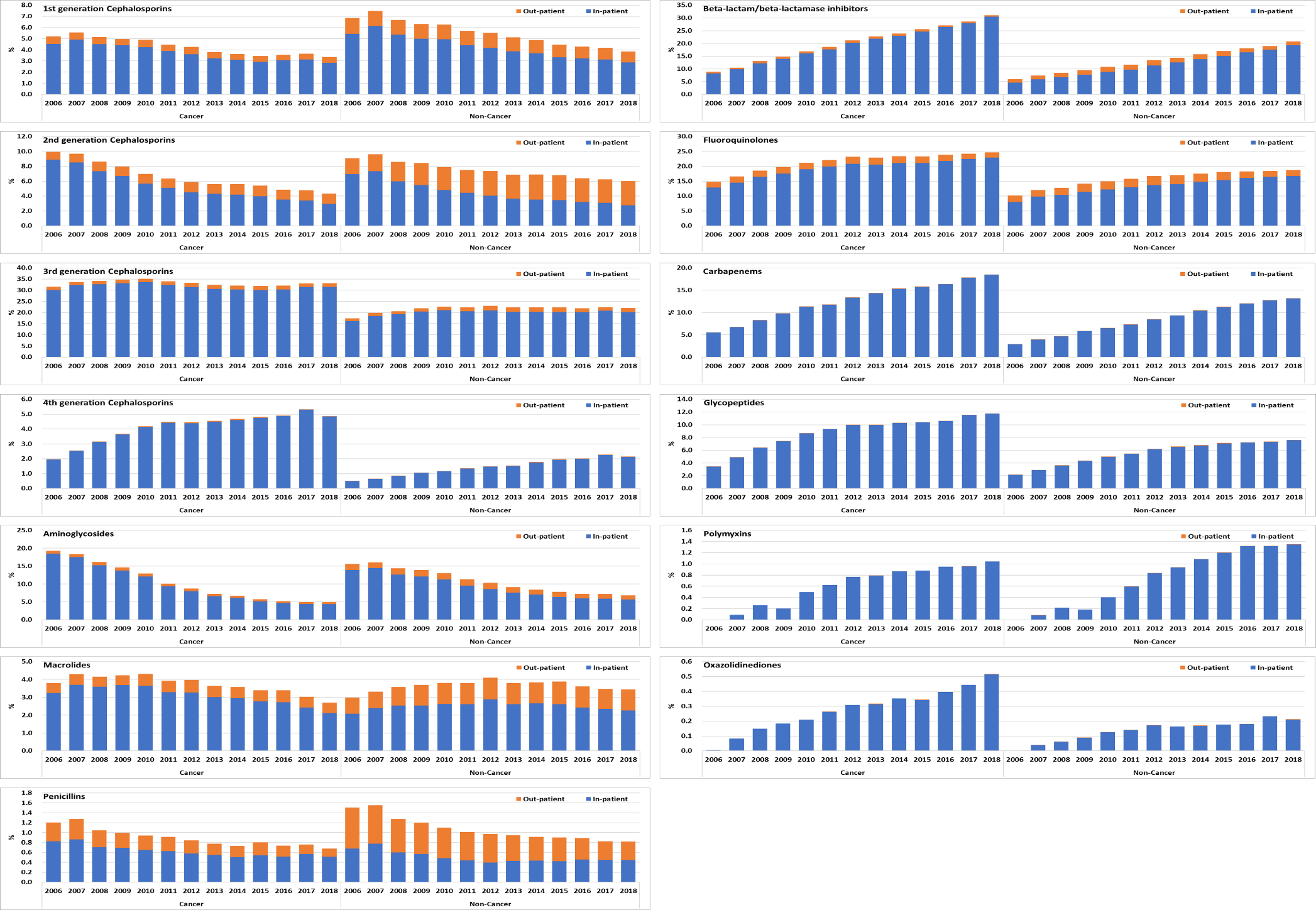
**Supplementary Figure 4**. Inpatient and outpatient prescription rates of antibiotic subclasses (%) in cancer and non-cancer decedents during the last month of life

**Supplementary Figure 5A**. Consumption rates of antibiotic subclasses (days-of-therapy/1000pt-days) among cancer decedents grouped by underlying cancer type compared to non-cancer decedents


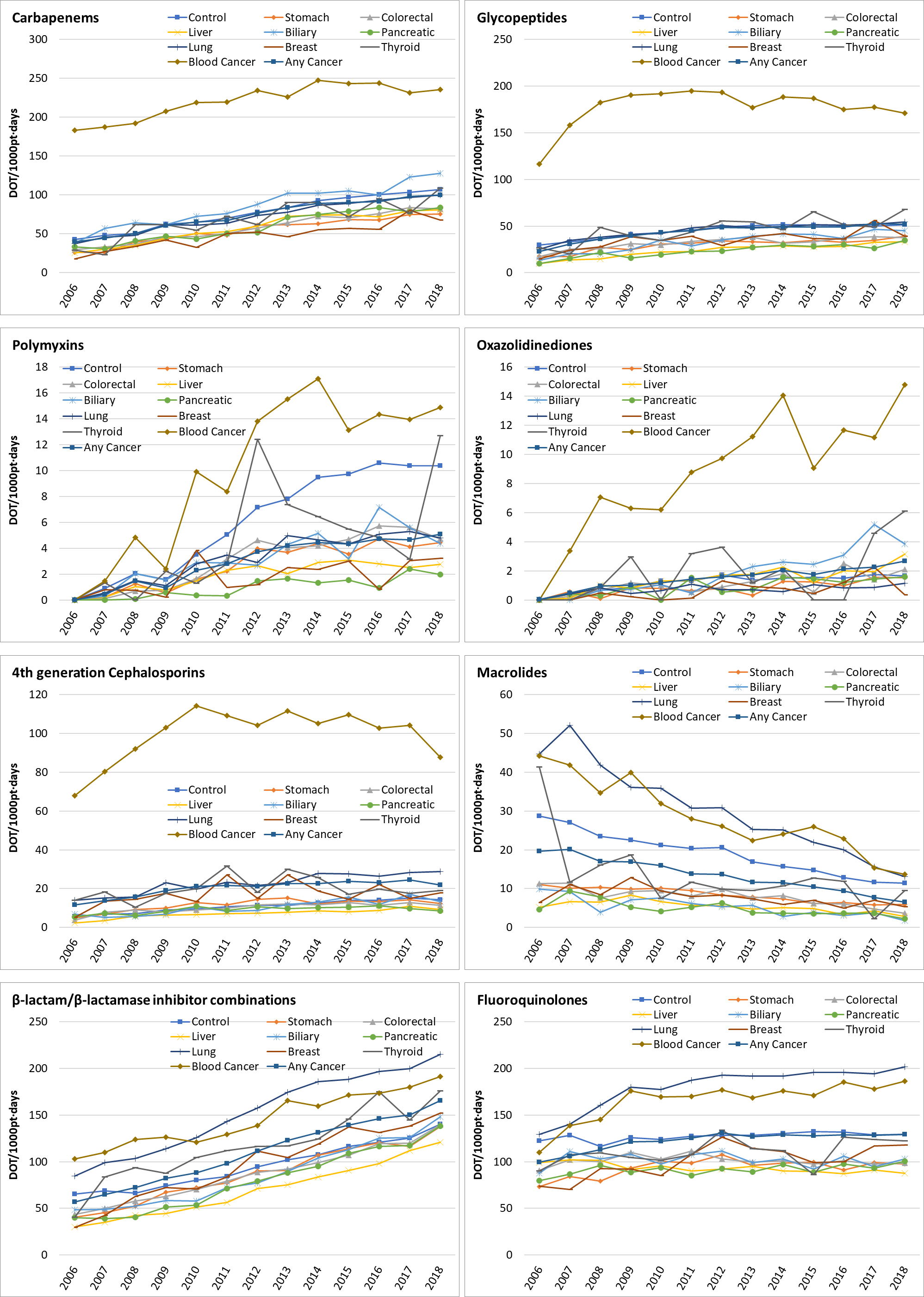


**Supplementary Figure 5B**. Prescription rates of antibiotic subclasses (%) among cancer decedents grouped by underlying cancer type compared to non-cancer decedents


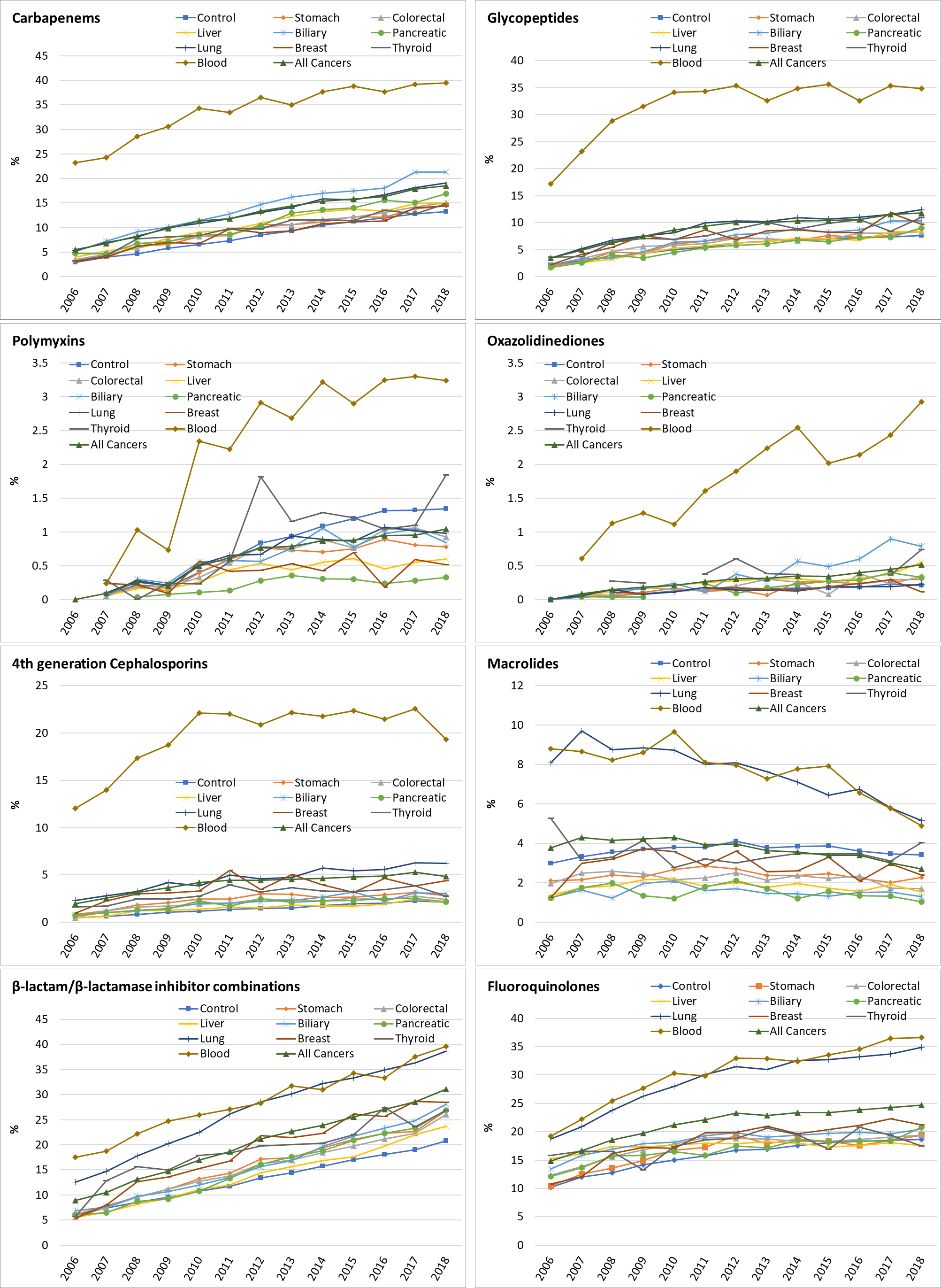


**Supplementary Figure 6**. Prescription rates of antibiotic subclasses in cancer and non-cancer decedents according to timespan preceding death (1 year, 6 months, and 1 month before death)


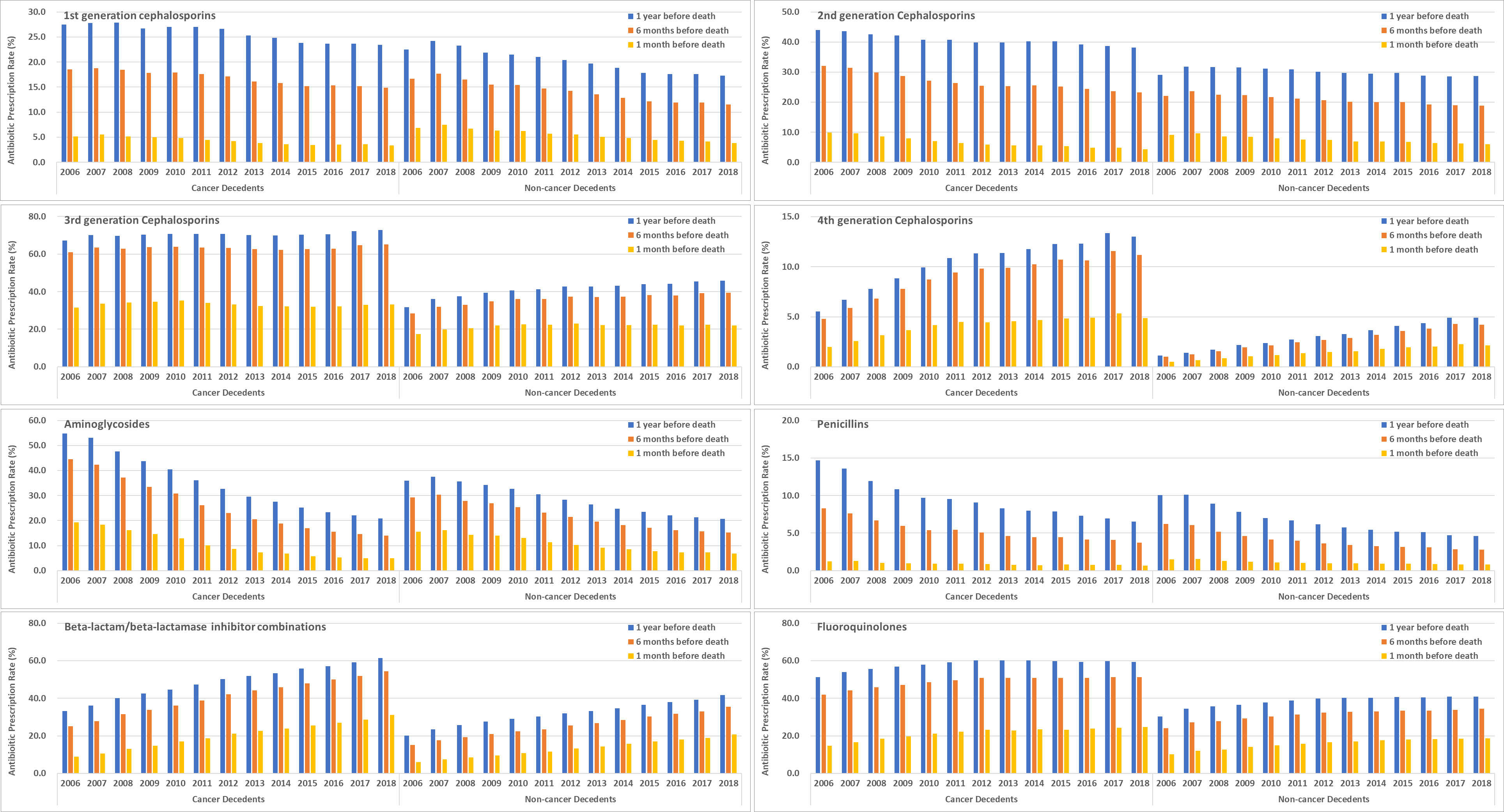


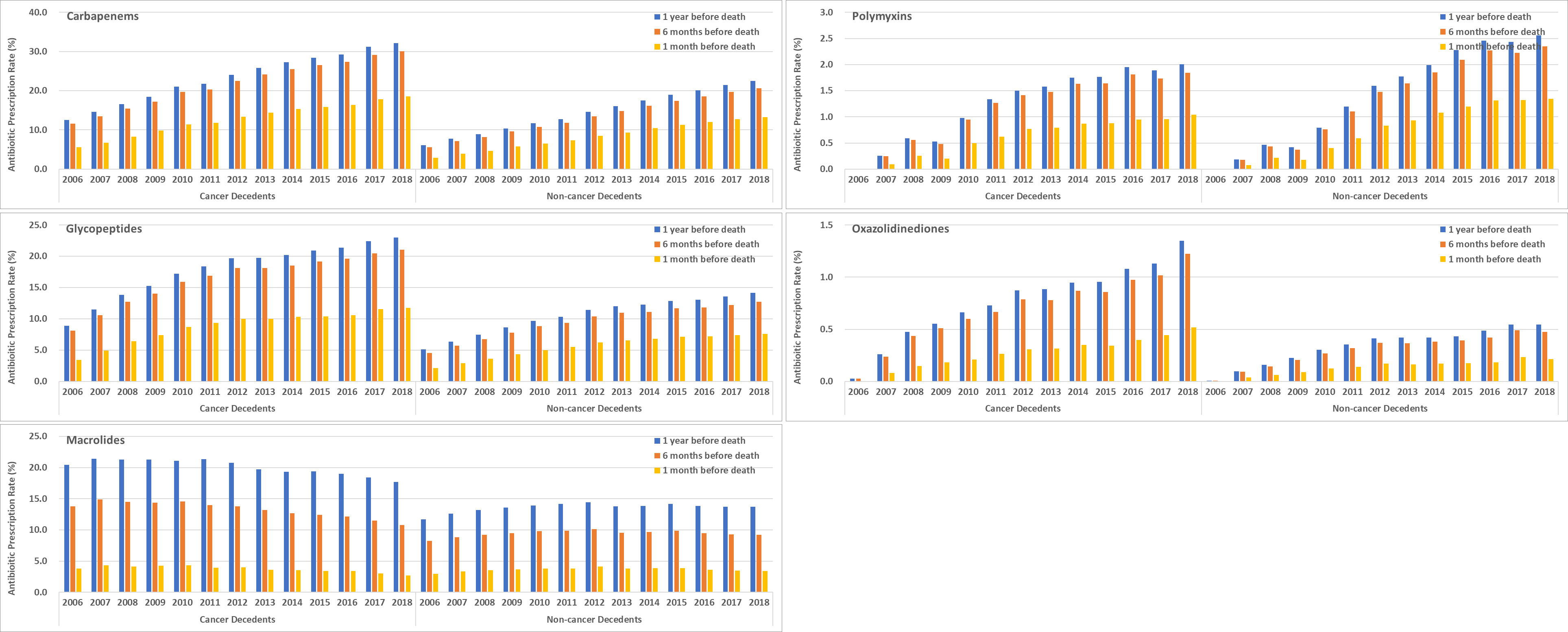


**Supplementary Figure 7**. Consumption rates of antibiotic subclasses in cancer and non-cancer decedents according to timespan preceding death (1 year, 6 months, and 1 month before death)


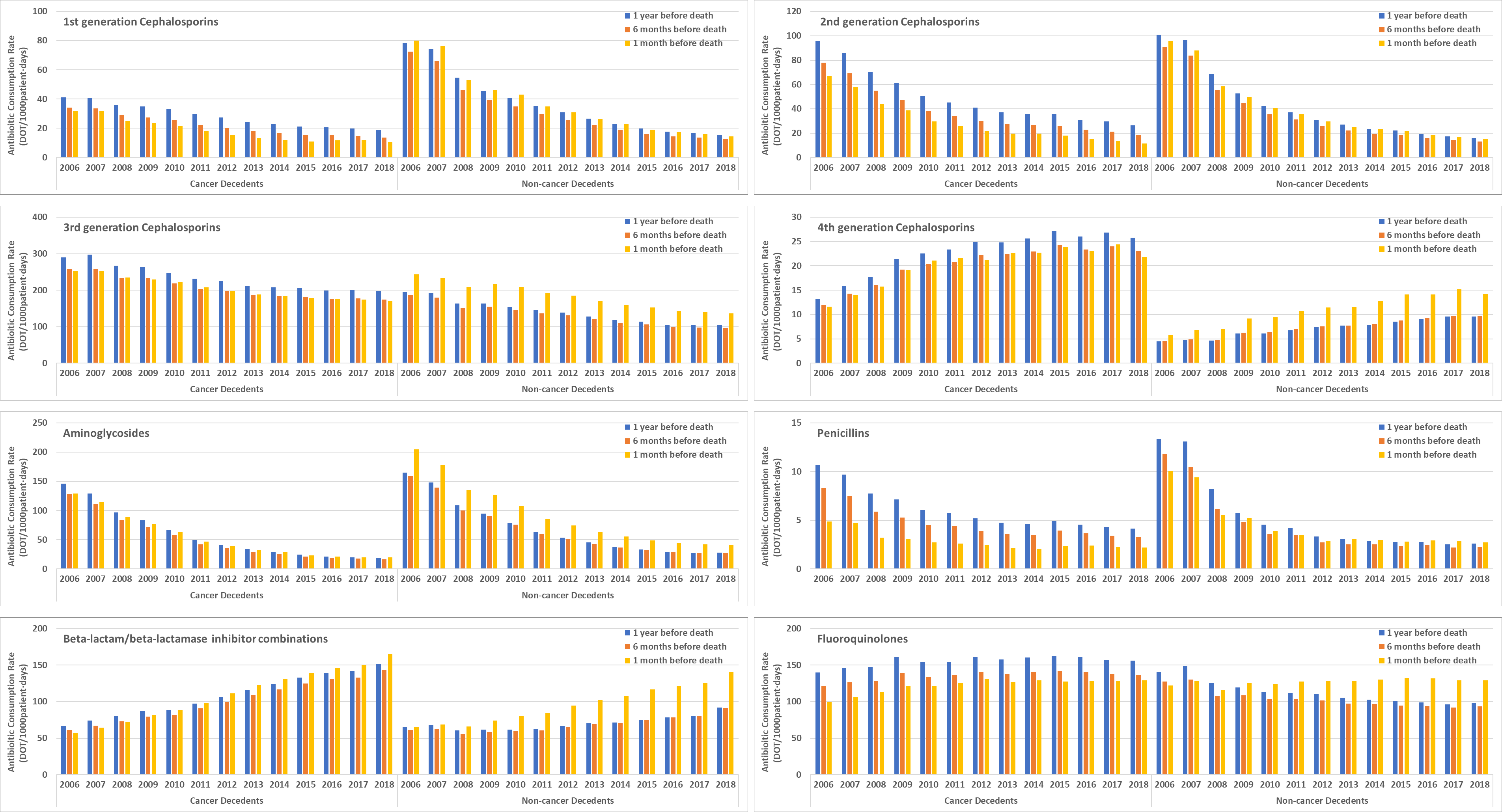


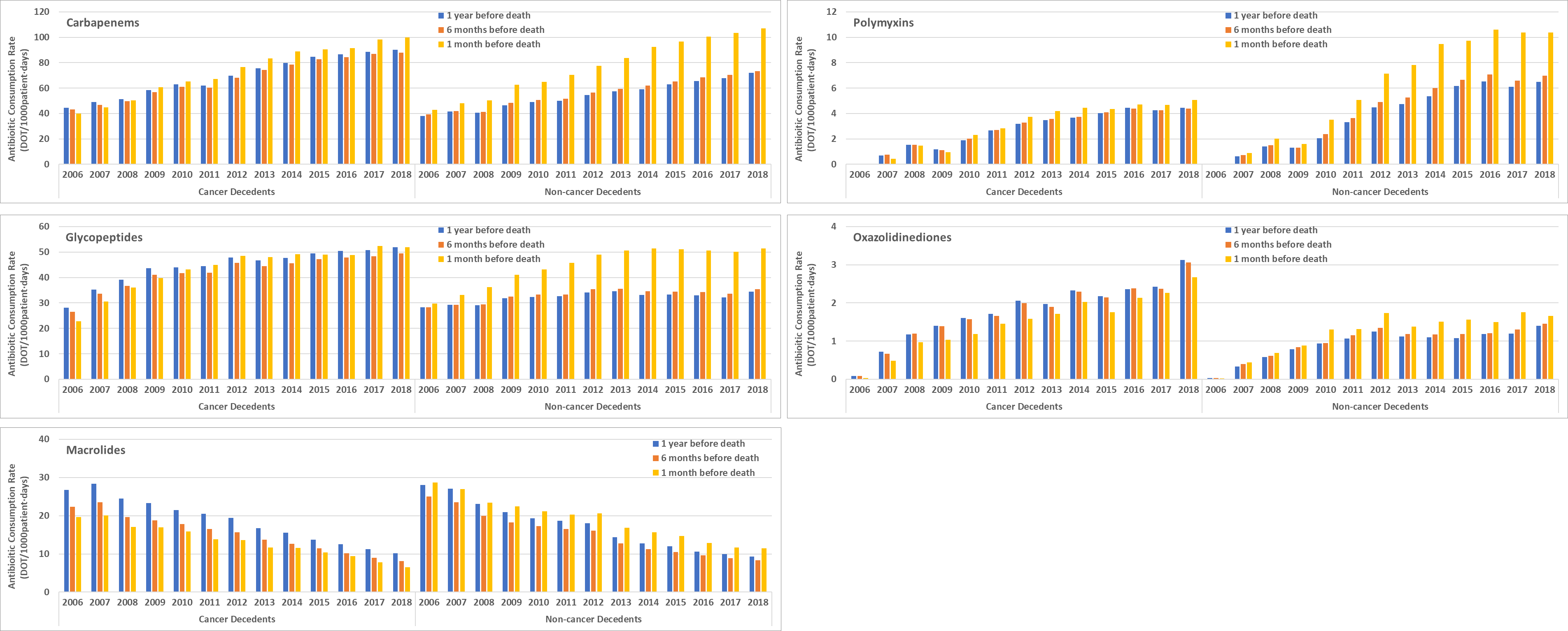


**Supplementary Table 1**. Temporal trends of antibiotic consumption rates in cancer and non-cancer decedents at different time intervals before death, 2006-2018

| Antibiotic subgroups |  | Temporal trends during 2006-2018 | | | | Relative change compared with 1 month before death | | | | | | | |
| --- | --- | --- | --- | --- | --- | --- | --- | --- | --- | --- | --- | --- | --- |
|  |  | 1 month before death | | | | 6 months | | | | 12 months | | | |
|  |  | Rate ratio | lower CI | upper CI | p-value | Rate ratio | lower CI | upper CI | p-value | Rate ratio | lower CI | upper CI | p-value |
| Overall | Non-Cancer | 0.977 | 0.977 | 0.977 | 0.0000 | 0.982 | 0.982 | 0.982 | 0.0000 | 0.978 | 0.978 | 0.978 | 0.0000 |
|  | Cancer | 0.996 | 0.996 | 0.996 | 0.0000 | 0.995 | 0.994 | 0.995 | 0.0000 | 0.992 | 0.992 | 0.992 | 0.0000 |
| 1st generation cephalosporins | Non-Cancer | 0.861 | 0.860 | 0.861 | 0.0000 | 0.996 | 0.996 | 0.997 | 0.0000 | 1.006 | 1.005 | 1.007 | 0.0000 |
|  | Cancer | 0.902 | 0.901 | 0.903 | 0.0000 | 1.020 | 1.019 | 1.022 | 0.0000 | 1.032 | 1.030 | 1.033 | 0.0000 |
| 2nd generation cephalosporins | Non-Cancer | 0.851 | 0.850 | 0.851 | 0.0000 | 0.990 | 0.989 | 0.990 | 0.0000 | 0.996 | 0.995 | 0.996 | 0.0000 |
|  | Cancer | 0.865 | 0.864 | 0.866 | 0.0000 | 1.027 | 1.026 | 1.028 | 0.0000 | 1.039 | 1.038 | 1.040 | 0.0000 |
| 3rd generation cephalosporins | Non-Cancer | 0.950 | 0.950 | 0.951 | 0.0000 | 0.993 | 0.993 | 0.993 | 0.0000 | 0.994 | 0.994 | 0.995 | 0.0000 |
|  | Cancer | 0.965 | 0.965 | 0.965 | 0.0000 | 1.000 | 0.999 | 1.000 | 0.3435 | 1.000 | 1.000 | 1.000 | 0.6508 |
| 4th generation cephalosporins | Non-Cancer | 1.071 | 1.070 | 1.072 | 0.0000 | 0.993 | 0.992 | 0.995 | 0.0000 | 0.995 | 0.994 | 0.996 | 0.0000 |
|  | Cancer | 1.039 | 1.038 | 1.040 | 0.0000 | 1.002 | 1.001 | 1.003 | 0.0037 | 1.002 | 1.001 | 1.004 | 0.0000 |
| Aminoglycosides | Non-Cancer | 0.864 | 0.864 | 0.865 | 0.0000 | 0.983 | 0.982 | 0.983 | 0.0000 | 0.980 | 0.979 | 0.980 | 0.0000 |
|  | Cancer | 0.836 | 0.836 | 0.837 | 0.0000 | 0.988 | 0.987 | 0.989 | 0.0000 | 0.987 | 0.986 | 0.988 | 0.0000 |
| β-lactam/β-lactamase inh combinations | Non-Cancer | 1.070 | 1.069 | 1.070 | 0.0000 | 0.972 | 0.971 | 0.972 | 0.0000 | 0.966 | 0.965 | 0.966 | 0.0000 |
|  | Cancer | 1.086 | 1.085 | 1.086 | 0.0000 | 0.987 | 0.987 | 0.988 | 0.0000 | 0.984 | 0.984 | 0.985 | 0.0000 |
| Carbapenems | Non-Cancer | 1.075 | 1.074 | 1.075 | 0.0000 | 0.980 | 0.980 | 0.981 | 0.0000 | 0.980 | 0.979 | 0.980 | 0.0000 |
|  | Cancer | 1.070 | 1.070 | 1.071 | 0.0000 | 0.991 | 0.990 | 0.991 | 0.0000 | 0.989 | 0.989 | 0.990 | 0.0000 |
| Fluoroquinolones | Non-Cancer | 1.006 | 1.006 | 1.006 | 0.0000 | 0.970 | 0.969 | 0.970 | 0.0000 | 0.963 | 0.963 | 0.963 | 0.0000 |
|  | Cancer | 1.015 | 1.015 | 1.016 | 0.0000 | 0.992 | 0.992 | 0.993 | 0.0000 | 0.991 | 0.991 | 0.992 | 0.0000 |
| Glycopeptides | Non-Cancer | 1.036 | 1.035 | 1.036 | 0.0000 | 0.978 | 0.978 | 0.979 | 0.0000 | 0.976 | 0.975 | 0.976 | 0.0000 |
|  | Cancer | 1.044 | 1.043 | 1.045 | 0.0000 | 0.991 | 0.990 | 0.992 | 0.0000 | 0.990 | 0.989 | 0.991 | 0.0000 |
| Macrolides | Non-Cancer | 0.925 | 0.925 | 0.926 | 0.0000 | 0.983 | 0.982 | 0.984 | 0.0000 | 0.982 | 0.981 | 0.983 | 0.0000 |
|  | Cancer | 0.917 | 0.916 | 0.919 | 0.0000 | 1.000 | 0.999 | 1.002 | 0.6947 | 1.002 | 1.000 | 1.003 | 0.0296 |
| Oxazolinediones | Non-Cancer | 1.092 | 1.088 | 1.096 | 0.0000 | 0.994 | 0.990 | 0.998 | 0.0030 | 0.994 | 0.990 | 0.998 | 0.0038 |
|  | Cancer | 1.127 | 1.122 | 1.132 | 0.0000 | 0.986 | 0.981 | 0.990 | 0.0000 | 0.985 | 0.980 | 0.990 | 0.0000 |
| Penicillins | Non-Cancer | 0.894 | 0.892 | 0.896 | 0.0000 | 0.963 | 0.961 | 0.965 | 0.0000 | 0.958 | 0.956 | 0.960 | 0.0000 |
|  | Cancer | 0.940 | 0.937 | 0.943 | 0.0000 | 0.989 | 0.985 | 0.992 | 0.0000 | 0.985 | 0.982 | 0.988 | 0.0000 |
| Polymyxins | Non-Cancer | 1.176 | 1.174 | 1.178 | 0.0000 | 0.990 | 0.988 | 0.992 | 0.0000 | 0.991 | 0.989 | 0.993 | 0.0000 |
|  | Cancer | 1.151 | 1.147 | 1.154 | 0.0000 | 0.991 | 0.987 | 0.994 | 0.0000 | 0.993 | 0.990 | 0.996 | 0.0001 |
| CI: Confidence interval, inh: inhibitor | | | | | | | | | | | | | |
